# Walker-256 Tumour-Induced Cachexia Altered Liver Metabolomic Profile and Function in Weanling and Adult Rats

**DOI:** 10.3390/metabo11120831

**Published:** 2021-12-01

**Authors:** Natália Angelo da Silva Miyaguti, Gabriela de Matuoka e Chiocchetti, Carla de Moraes Salgado, Leisa Lopes-Aguiar, Lais Rosa Viana, Lea Blanchard, Rogério Willians dos Santos, Maria Cristina Cintra Gomes-Marcondes

**Affiliations:** 1 Laboratory of Nutrition and Cancer, Department of Structural and Functional Biology, Biology Institute, University of Campinas (UNICAMP), Rua Monteiro Lobato, 255, Campinas 13083862, SP, Brazil; gmchiocchetti@gmail.com (G.d.M.e.C.); carlamsalgado@gmail.com (C.d.M.S.); leisaaguiar@yahoo.com.br (L.L.-A.); lala.viana311088@gmail.com (L.R.V.); lelea_53@hotmail.fr (L.B.); rowisa@unicamp.br (R.W.d.S.); 2 Biology Department, Université d’Angers, 4900 Angers, France

**Keywords:** cachexia, experimental model, liver, metabolomics

## Abstract

Cancer cachexia occurs in up to 85% of advanced cancer patients, affecting different tissues and organs, mainly the liver, which plays a central role in body metabolism control. However, liver responses to cancer cachexia progression are still poorly understood. Considering the possible different challenges provided by the rodent’s phase of life and the cachexia progression, we evaluated the liver metabolic alterations affected by Walker-256 tumour growth in weanling and young-adult rats. For this, we applied a metabolomics approach associated with protein and gene expression analyses. Higher amino acid levels and impaired glucose metabolism were important features in tumour-bearing animals’ liver tissue. The weanling hosts had more pronounced cachexia, with higher carcass spoliation, liver lipid metabolism and impaired CII and CIV mitochondrial complexes. The liver alterations in young adult tumour-bearing rats were related to energy status and nucleotide metabolites, such as uridine, NAD+, xanthosine, hypoxanthine and inosine. In conclusion, the Walker-256 tumour-induced cachexia impaired liver metabolism, being more severe in the weanling hosts. Further studies are needed to correlate these changes in the preclinical model, which can be correlated to the clinical features of cancer cachexia, allowing for a translational potential involving the liver function and its responses to potential treatments.

## 1. Introduction

Cancer cachexia is marked by a disrupted metabolism, mainly affecting the skeletal muscle, accompanied or not by a loss of body fat mass, which occurs in up to 85% of advanced cancer patients, being responsible for at least 22% of cancer-related deaths [1,2]. It is also considered a multiorgan syndrome, affecting other organs and tissues, including damaging the hepatic system [1]. During cancer cachexia, the liver shows higher activity and also contributes to chronic cancer-associated inflammation. However, its role in the disease is still poorly understood [1,3]. The Cori cycle occurs in the liver and is responsible for high energy dissipation rates, being responsible for exacerbating the energy waste in cancer cachexia conditions [4]. In cachectic rats, an excess of cardiolipin leads to a reduced mitochondrial oxidative phosphorylation, which exacerbates the energy-wasting [5]. Different from skeletal muscle, liver protein synthesis is upregulated, increasing the production of the acute phase reactants, such as the C-reactive protein (CRP) [6], which is associated with a poor prognosis in cachectic patients [7]. Additionally, hepatic steatosis, another liver metabolism alteration, and liver-muscle crosstalk have been suggested to worsen the cancer cachexia wasting, showing the importance of more studies on this topic [3,8].

Metabolomics is a valuable approach to studying highly complex systems, verifying the ending results of metabolism. It allows the assessment of low molecular weight components in biosamples, reflecting the phenotype derived from gene-environment interactions of a response at a given time [9,10]. The metabolic profiling of biological fluids or tissue samples provides information to help in disease diagnosis, staging and prognosis, as well as to understand drug interactions and side effects [11,12]. Many studies are emerging in the field of cancer cachexia metabolomics [13,14]. Our previous ^1^H-NMR-based study showed serum metabolite alterations associated with muscle metabolic pathways related to energy production and protein breakdown in Walker-256 tumour-bearing animals [15,16]. We also found metabolic changes in skeletal muscle, showing altered metabolites mainly related to increased levels of amino acids and altered energetic metabolism, suggesting an expressive catabolic process and energy production in the youngest host, depicting differences between muscle responses depending on the host developmental stages [17].

Metabolomic analysis of liver tissue from C26 tumour-bearing animals showed perturbations on energy production and increased liver glucose and glycogen depletion [18]. Likewise, recent studies have reported liver metabolism impairment [19] and mitochondrial dysfunction [20] in this same experimental model. Pötgens et al. reported that liver glycolysis and gluconeogenesis were impaired, and the CRP production was augmented with a higher amino acid uptake by this organ, followed by reduction of carnitine levels and lower activation of the phosphatidylcholine pathway, possibly contributing to liver steatosis [19]. Despite these studies, the relationship between liver metabolism and its impairment in cancer cachexia is still not fully clarified.

Considering the increasing need for knowledge of the hepatic metabolism responses to the onset of cancer cachexia and the challenges of liver responses to diseases and medicines during different stages of life [21], we proposed to evaluate the alterations in metabolic liver profile in Walker-256 tumour-bearing rats in the weanling and adult stages of life.

## 2. Results

### 2.1. Morphometric and Serum Parameters Indicate Cachexia Installation in Both Weanling and Young Adult Groups

At the beginning of the experiment, the initial body weight showed no difference between both weanling groups [(weanling tumour-bearing group (WW) = weanling control group (WC)] and also for both adult groups [(adult tumour-bearing group (AW) = adult control group (AC)] (Table S1). However, after the tumour growth, we observed a negative impact in host weight, as both tumour-bearing groups had a significant decrease in carcass weight (WW < WC, ≈31% of reduction; AW < AC, ≈13% of reduction; where tumour and age factors were significant; Table S1). In relation to muscle spoliation, only the WW group had a reduced gastrocnemius muscle relative weight compared to its respective control (WW < WC, ≈41% reduction; being age, tumour, and interaction factors significant for this parameter; Table S1). Meanwhile, the tumour growth had no impact on the relative liver weight; nevertheless, the age factor was significant (Table S1). Also, the relative tumour weights were similar between the WW vs. AW groups (Table S1). As an indicator of cachexia installation, the cachexia index differed between WW vs. AW groups (Table S1), showing an intense spoliation, especially in the weanling tumour-bearing group (WW, ≈51% compared to AW, ≈21% of cachexia index).

Regarding serum parameters, as expected in cachectic animals, glucose levels decreased in both tumour-bearing groups (≈49% less in WW vs. WC; and ≈30% less in AW vs. AC; Table S1). This reduction was more expressive in the WW group (33% lower) than in AW; tumour and age factors were responsible for this significance (Table S1). The same reduction pattern occurred for total protein (≈25% decreased, WW < WC; ≈11% reduced, AW < AC; and ≈24%, WW <AW; showing that tumour, age, and interaction factors were significant; Table S1) and albumin contents (reduction of ≈26%, WW < WC; ≈18%, AW < AC; and ≈14% less, WW < AW; in which tumour and age factors were significant; Table S1).

### 2.2. Liver Metabolic Differences Provided by Tumour Growth in Different Stages of Life

For liver metabolomics analysis, 49 metabolites were assigned, and a representative spectrum of the metabolite assignment is presented in Figure S1. To guide the discussion, we manually separated the metabolites into five groups: amino acids, lipid metabolism-related metabolites, energy status and nucleotide-related metabolites, glucose metabolism and other metabolites. Betaine was included in the amino acid category, as a modified amino acid and lipid metabolism-related category, as a metabolite that participates in glycerophospholipid metabolism. Moreover, we included the liver glycogen content in this metabolite grouping.

**Figure 1:**
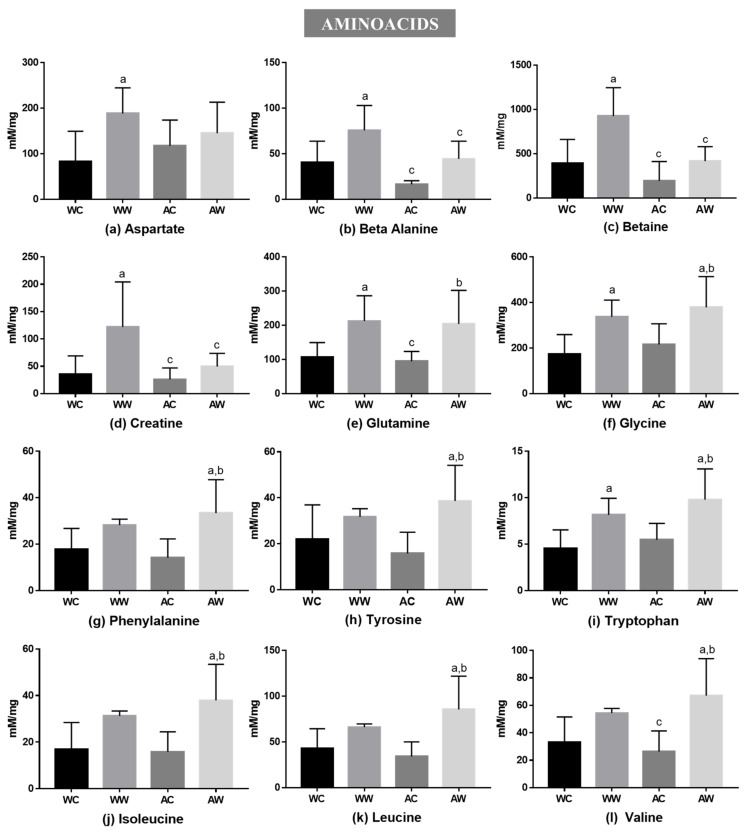
Liver metabolites grouped into amino acids in weanling and adult control and tumour-bearing animals: (**a**) aspartate; (**b**) beta-alanine; (**c**) betaine; (**d**) creatine; (**e**) glutamine; (**f**) glycine; (**g**) phenylalanine; (**h**) tyrosine; (**i**) tryptophan and BCAAs: (**j**) leucine; (**k**) leucine; (**l**) valine. Legend: WC, weanling control group (*n* = 7); WW, weanling tumour-bearing group (*n* = 10); AC, adult control group (*n* = 6); AW, adult tumour-bearing group (*n* = 8). Data were expressed as mean ± standard deviation (SD), millimolar/milligram of liver tissue. Data were analysed by two-way ANOVA and corrected for multiple comparisons by the post hoc test Bonferroni. ^a^ represents differences from the WC group ^b^ indicates differences from AC group and ^c^ indicates differences from the WW group. Differences were significant when *p* < 0.05.

In the amino acid category, 12 metabolites presented differences in liver tissue among the groups (Figure 1). The tumour factor significantly affected all these metabolites; the age factor was significant only for betaine and creatine (Table S2). Comparing the weanling groups (WW vs. WC), the tumour growth increased the levels of seven amino acids (WW > WC): aspartate, beta-alanine, betaine, creatine, glutamine, glycine and tryptophan (Figure 1). In addition, comparing the adult groups (AW vs. AC), eight amino acids increased (AW > AC): glutamine, glycine, phenylalanine, tyrosine, tryptophan, isoleucine, leucine and valine. Regarding tumour growth, only glutamine and tryptophan were increased independent of animal age. Comparing the tumour-bearing groups (WW vs. AW), beta-alanine, betaine and creatine concentrations were enhanced in the WW group compared to the AW (the WW group was over 2-fold higher than AW for these amino acids) (Figure 1).

**Figure 2:**
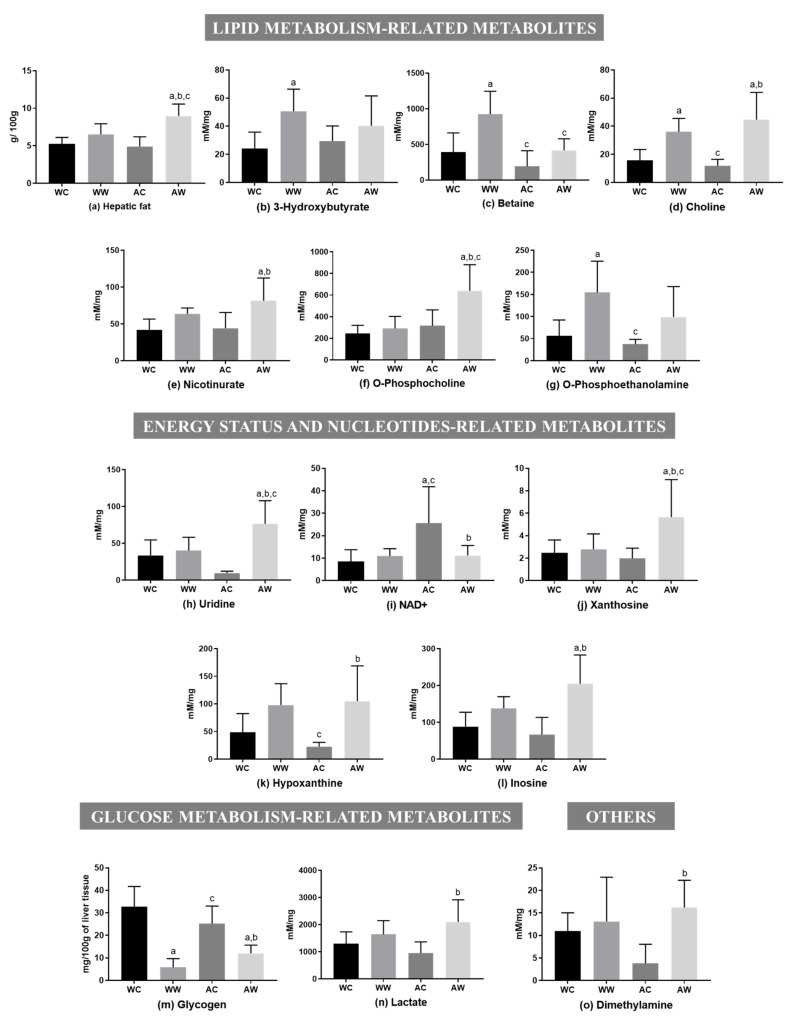
Liver metabolites in weanling and adult control and tumour-bearing animals. Lipid metabolism-related metabolites: (**a**) hepatic fat; (**b**) 3-hydroxybutyrate; (**c**) betaine; (**d**) choline; (**e**) nicotinurate; (**f**) O-phosphocholine; (**g**) O-phosphoethanolamine. Energy status and nucleotide-related metabolites: (**h**) uridine; (**i**) NAD+; (**j**) xanthosine; (**k**) hypoxanthine (**l**) inosine. Glucose metabolism-related metabolites: (**m**) glycogen; (**n**) lactate. Others: (**o**) dimethylamine. Legend: WC, weanling control group (*n* = 7); WW, weanling tumour-bearing group (*n* = 10); AC, adult control group (*n* = 6); AW, adult tumour-bearing group (*n* = 8). Data were expressed as mean ± standard deviation (SD), millimolar/milligram of liver tissue or mg/100 g of liver tissue for glycogen content. Data were analysed by two-way ANOVA and corrected for multiple comparisons by the post hoc test Bonferroni. ^a^ represents differences from the WC group, ^b^ indicates differences from AC group and ^c^ indicates differences from the WW group. Differences were significant when *p* < 0.05.

The total liver fat was increased in the AW group in relation to the AC and WW groups (AW > AC, ≈45% of increase; AW > WW, ≈27% of increase; tumour, age and interaction were significant; Figure 2a). Moreover, in relation to lipid metabolism-related metabolites, five of them presented differences among the groups (Figure 2). The tumour factor had a significant effect on all these metabolites, being the age factor significant only for betaine. For O-phosphocholine, tumour, age and interaction had a significant effect (Table S2). Comparing the weanling groups, we verified that tumour evolution raised the content of 3-hydroxybutyrate, betaine and choline (WW > WC) (Figure 2). In addition, comparing the adult groups, three metabolites (choline, nicotinurate and O-phosphocholine) increased due to tumour growth (AW > AC). In this way, regarding tumour growth, only choline increased independently of group age. Comparing the tumour-bearing groups (WW vs. AW), betaine (WW was 2.2-fold higher than AW) and O-phosphocholine (WW was ≈54% lower than AW) concentrations differed in the WW group in relation to the AW group (Figure 2).

Five liver metabolites showed differences among the groups for the energy status and nucleotide-related metabolites (Figure 2). The tumour and interaction age/tumour factors significantly impacted all these metabolites, except for hypoxanthine, in which the interaction factor was not significant. In addition, the age factor had significant importance only for NAD+ (Table S2). Thus, no differences were found between the weanling groups (WW = WC). However, all metabolites showed differences when comparing both adult groups (AW vs. AC). Uridine, xanthosine, hypoxanthine and inosine contents increased in the AW group (AW > AC), but NAD+ was reduced (AW < AC). Comparing both tumour-bearing groups (WW vs. AW), uridine and xanthosine content decreased in the WW group compared to the AW group (WW was ≈48% lower than AW) (Figure 2).

Two metabolites were assigned for glucose metabolism-related metabolites, in which the tumour factor had a significant effect, and also the interaction factor significantly led to glycogen depletion (Table S2). Hepatic glycogen content was reduced in both tumour-bearing groups in comparison to respective control groups [(WW < WC (≈98% less); AW < AC (≈52% less)], and lactate was only increased in the adult group, in which the AW group had 2.2-fold higher levels than the AC group. O-phosphoethanolamine increased in WW in relation to WC (WW > WC), and dimethylamine increased in AW compared to AC (AW > AC); both metabolites were grouped into the “other category”, and the tumour factor had a significant effect (Table S2).

### 2.3. Liver Differences in Protein and Gene Expression Modified by Tumour Growth in Different Stages of Life

**Figure 3:**
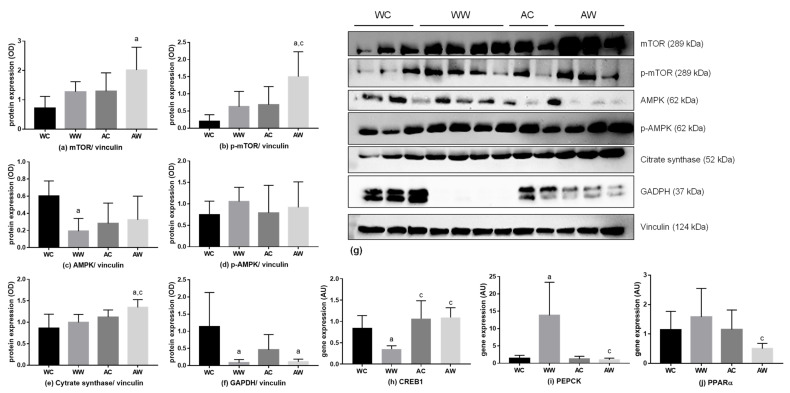
Protein and gene expression in the liver of weanling and adult control and tumour-bearing animals. Western blot analysis from: (**a**) total mTOR; (**b**) phosphorylated mTOR; (**c**) total AMPK; (**d**) phosphorylated AMPK; (**e**) citrate synthase; (**f**) GAPDH; (**g**). The western blot images are representative of the total analysis of all animals in each group. Rt-PCR analysis for gene relative expression of: (**h**) *CREB1*, (**i**) *PEPCK* and (**j**) *PPAR α*. Legend: WC, weanling control group (*n* = 7); WW, weanling tumour-bearing group (*n* = 10); AC, adult control group (*n* = 6); AW, adult tumour-bearing group (*n* = 8). Data were expressed as mean ± standard deviation (SD). The units used were as follows: (**a**–**f**): protein/vinculin protein expression (optical density, OD—western blot image) and (**h**–**j**): relative protein expression (rt-PCR; expressed as arbitrary unit- AU). Data were analysed by two-way ANOVA and corrected for multiple comparisons by the post hoc test Bonferroni. ^a^ represents differences from the WC group and ^c^ indicates differences from the WW group. Differences were significant when *p* < 0.05.

Some protein expressions related to liver metabolism were evaluated, and the results are available in Table S3. The total mechanistic target of rapamycin (mTOR) protein expression had no change among the comparisons made (WC = WW; AC = AW; WW = AW, Figure 3a), but both tumour and age factors were significant for this protein (Table S3). Additionally, the protein expression of phosphorylated mTOR was higher in AW when compared to the WW groups (AW > WW, Figure 3b), also presenting a significant effect by age and tumour factors (Table S3). The protein expression of total AMP-activated protein kinase (AMPK) decreased in WW when compared to respective control (WW < WC, Figure 3c), being the interaction significant for this difference (Table S3). However, phosphorylated AMPK remained unchanged in all groups (Figure 3d). Regarding the citrate synthase expression, there was no change in WW animals when compared to their control. However, this protein expression increased in the AW group in comparison to AC and WW (AW > AC and AW > WW, Figure 3e), in which the age factor had a significant impact (Table S3). In relation to the glyceraldehyde-3-phosphate dehydrogenase (GAPDH), the expression levels decreased in both weanling and adult tumour-bearing groups (WW < WC; AW < AC, Figure 3f), with the tumour acting as a significant factor for this difference (Table S3).

Also, to show gene expression related to the main analysed proteins, Table S4 presents all data of the rt-PCR, showing all analysed genes. The *CAMP responsive element binding protein 1* (*CREB1*) was evaluated, and the age and the interaction between age/tumour factors were significant (Table S4) and the relative gene expression decreased in WW *vs* AW (WW < AW; Figure 3h). Moreover, the relative expression of *phosphoenolpyruvate carboxykinase (PEPCK)* was increased in WW when compared to the respective control (WW > WC; Figure 3i). Comparing both tumour-bearing groups, the *PEPCK* relative expression was reduced in the AW group in relation to the WW group (WW > AW). Also, the age, tumour and interaction were significant for this result (Table S4). For *peroxisome proliferator-activated receptor alpha* (*PPAR α)* expression, comparing both tumour-bearing groups, the AW relative expression was diminished in relation to WW (WW < AW, Figure 3j).

**Figure 4:**
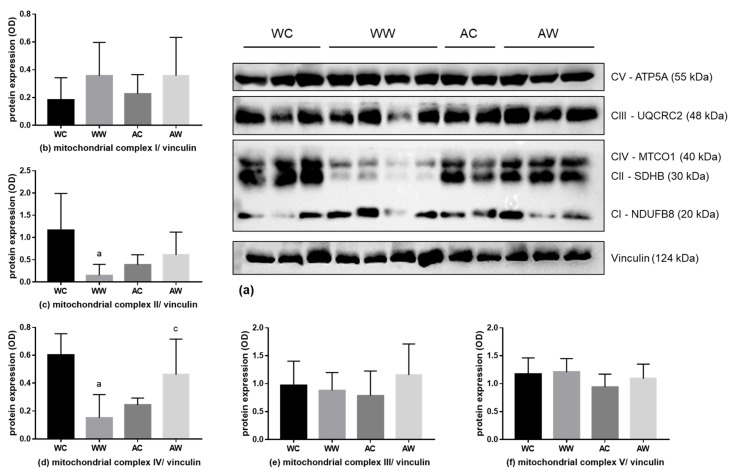
Liver protein expression of the mitochondrial respiratory complex. (**a**) Western blot images are a representative image of the mitochondrial respiratory complexes and the loading control vinculin of the total analysis of all animals in each group. Analyses of the protein expression of mitochondrial respiratory complexes; the subunits protein expression are presented in (**b**) V subunit; (**c**) III subunit; (**d**) IV subunit; (**e**) II subunit; and (**f**) I subunit. For subunit details, please see the Material and Methods section. Legend: WC, weanling control group (*n* = 7); WW, weanling tumour-bearing group (*n* = 10); AC, adult control group (*n* = 6); AW, adult tumour-bearing group (*n* = 8). Data were expressed as mean ± standard deviation (SD). The units used were protein/vinculin protein expression (optical density, OD—western blot image). Data were analysed by two-way ANOVA and corrected for multiple comparisons by the post hoc test Bonferroni. ^a^ represents differences from the WC group and ^c^ indicates differences from the WW group. Differences were significant when *p* < 0.05.

Regarding the mitochondrial liver content and activity, we also evaluated CI–V respiratory chain subunits (Figure 4a). The expression of the CII subunit was diminished in WW animals compared to the respective control (WW < WC, Figure 4c). The expression of the CIV subunit was also lower in the WW group when compared to the WC and AW groups (WW < WC; WW < AW, Figure 4d). The interaction factor was significant for CII and CIV subunit differences (Table S3). The CI, CIII and CV subunits remained unchanged in all groups (Figure 4b,e,f, respectively).

## 3. Discussion

The liver is also severely compromised, in addition to the widely studied loss of skeletal muscle, during the progression of cancer cachexia. However, the hepatic role and its alterations in this disease are still poorly understood [1,3]. Cancer cachexia is a clinical need that is not totally met; thereby, preclinical studies designed with the correct choice of the animal model are of extreme importance to elucidate the mechanisms and treatments for this syndrome [22,23]. In this context, it is important to consider the differences in liver metabolism responses to diseases and drug administration in accordance with the rodent phase of life [21]. Therefore, here, we evaluated the impact of Walker-256 tumours on changes in liver function, according to disturbance of hepatic metabolism and metabolomic profile during tumour development in weanling and young adult rats.

The role of the liver during cancer-cachexia has aroused increasing interest, with evidence suggesting that it is closely involved in cachexia-induced body weight loss and muscle atrophy [24,25]. Following literature data, here, we found a diminished carcass weight added to a reduction in serum albumin and total protein levels, features related to the cachexia process. These results were similar to previous studies using the same experimental model [26,27,28]. Of note, these alterations happened in both tumour-bearing groups but were more severe in WW animals, likely suggesting a higher spoliation in younger hosts, corroborating our previous report [17]. Although we did not find differences in relative liver weight in the present study, we found an altered liver metabolomic profile, gene and protein expressions imposed by the tumour growth, which were differentially affected by life stage in tumour-bearing animals.

During cancer cachexia, the liver plays a central role in regulating the systemic metabolic response to nutritional deficit [3]. Regarding glucose metabolism, we found diminished serum glucose in parallel to a depletion in the liver glycogen stores in both tumour-bearing groups. In this way, to provide glucose attempting to sustain the host metabolism, this intense glycogenolysis, which is not maintaining the glycaemia, is associated with an increased gluconeogenesis process [29]. In muscle wasting during cachexia, amino acids are mobilised and released from skeletal muscle, providing higher availability of precursors for gluconeogenesis [29,30]. Moreover, liver gluconeogenesis could also be supported by substrates provided from lipolysis (glycerol) and other sources, such as lactate derived from muscle and tumour tissue [25]. In the present study, we found an increase in amino acid content in the liver tissue of tumour-bearing animals. These results are in accordance with a liver metabolomic study using the C26 cachexia murine model [19] and possibly indicate that the uptake these substrates is derived from muscle protein breakdown. Nevertheless, among the twelve amino acids found here, only the content of glutamine, tryptophan and glycine increased in both tumour-bearing groups, requiring further studies to better understand this differential increase in amino acids between different stages of life.

The liver protein synthesis may also be affected during cancer cachexia. It was shown that tumour burden increases protein synthesis rates in the liver of cachectic APCM^in/+^ tumour-bearing mice [31]. In this way, as mTOR is activated during protein synthesis [29], the increased p-mTOR/mTOR ratio (1.4-fold in AW and 1.8-fold in WW rats) likely suggests a higher liver activity in these tumour-bearing animals. Supporting this increased activity, during muscle spoliation, the liver utilises the free amino acids available to synthesise proteins, especially the acute-phase proteins, such as the C-reactive protein [32]. However, we did not find any change in C-reactive protein gene expression in either tumour-bearing animal group. In addition to this point, similar to results from Pötgens et al. [12], we also found a 5-fold higher pAMPK/AMPK ratio in WW, which is a hallmark of a low-energy state in the liver. In our results, the activated mTOR likely led to an enhanced in hepatic activity, which in the case of cachexia is likely associated with higher liver AMPK [29], possibly favouring increased gluconeogenesis in these animals [29]. Corroborating these findings, a study with the C26 model also showed higher activation of liver’s AMPK expression, in which the phosphorylation of AMPK increased in body weight stable tumour-bearing mice and remained elevated in moderate cachexia [19]. In addition, gluconeogenesis is regulated by the availability of gluconeogenic substrates [29]. Thus, the main glucogenic and some ketogenic metabolites found increased in both tumour-bearing animals, such as alanine, lactate and other amino acids, such as glutamine, glycine, tryptophan, isoleucine and phenylalanine, could give their carbon skeletons to be transformed into new glucose [29,33,34], reinforcing the increased gluconeogenesis process.

Otherwise, the CREB1 expression, a gluconeogenic transcription factor phosphorylated by protein kinase A (PKA), which stimulates the expression of PEPCK, G6P and PGC1α [35], unexpectedly decreased in WW rats, which is curiously followed by an augmented expression of liver’ PEPCK (the main gluconeogenic enzyme) in these animals. In this way, increased activation of gluconeogenesis represented a higher energy expenditure by the Cori cycle, likely leading to an intense glucose demand, confirmed by the higher host spoliation in the WW group. On the other hand, we reinforced that the liver function changed in both tumour-bearing animals since the decrease in GAPDH protein expression might reflect the diminished activity of the glycolytic process, even with no changes in GAPDH gene expression but similar liver content of NAD+ in WW, or significantly decreased in the AW group. These results require further investigations since the main protein keys found here were related to the increased gluconeogenesis process.

As mentioned above, the liver energy metabolism is deeply affected in cancer cachexia [36]. It is known that betaine and choline, in addition to being related to lipid metabolism, are also involved in mitochondrial activity and function [37,38,39]. Recently, Lee suggested that betaine is a positive regulator of mitochondrial respiration in betaine-treated H2.35 cells, an epithelial-like cell line derived from a primary hepatocyte culture, showing an upregulation of mitochondrial respiration and cytochrome c oxidase activity added to increased mitochondrial membrane potential and cellular energy levels [37]. However, our results showed that the tumour’s effect in enhancing the content of these two metabolites in the liver might likely be related to altered liver mitochondrial function. Corroborating this fact, in C26 tumour-bearing mice, impaired liver mitochondrial dynamics has been reported to contribute to altered bioenergetics and further impaired skeletal muscle respiration [20]. Halle et al. described that uncoupling of mitochondrial oxidative phosphorylation could worsen the energy inefficiency contributing to body weight loss [20]. In this way, the tumour evolution probably affected the liver mitochondrial activity by enhancing de novo synthesis of choline and betaine as well, using phosphocholine (despite being increased only in the AW group). This new synthesis occurs even under the lower nutritional provision, as found by the anorexia presented in tumour-bearing mice [17]. Therefore, even at increased levels of choline or betaine, there was a decrease in the enzymes CII and CIII of the OXPHOS mitochondrial complex, suggesting the diminished mitochondrial activity in the liver of the WW group. Similarly, Khamoui et al. [40] found decreased protein expression of mitochondrial complex II and III in moderate and severe body weight loss in C26-tumour-bearing mice. Moreover, the changes found in GAPDH and CREB1 protein expression could be related to a decreased glycolytic process, tricarboxilic acid cycle and increased gluconeogenesis [29,40,41,42], respectively, probably reflecting a reduced liver cell respiration and their inability to maintain ATP production. These alterations likely implied mitochondrial dysfunction, contributing to the pronounced cachexia described in this WW group.

Liver lipid metabolism is also disrupted by tumour evolution, which is linked with the mentioned increased content of choline and betaine, as well as the O-phosphocholine and nicotinurate augmented only in AW animals, possibly reflected in liver lipid accumulation, as denoted by the higher liver lipid content. Additionally, these points could be associated with no change in WW, or even a decrease in the expression of PPAR-α, in the AW group [43,44], which is likely related to hepatic steatosis [45,46] or enhanced lipid oxidation or ketogenesis, suggested by increased 3-hydroxybutyrate in the WW group. These results were similar to the one found by Pötgens’ [19], in which the expression levels of PPAR, among other genes involved in mitochondrial fatty acid oxidation, were unchanged in C26 tumour-bearing mice. In addition, corroborating this point, we found increased content in uridine, xanthosine, hypoxanthine and inosine, metabolites related to purine and pyrimidine metabolism, in both tumour-bearing groups regardless of animal age. These alterations were likely related to changes in nucleotide metabolism and degradation [47]. In fact, as shown by Pillwein et al., these metabolites were directly affected in rat hepatoma, showing compromised liver function [48]. However, AW animals had an important hepatic impairment related to these metabolites, requiring further exploration of tumour effects that affect this pathway in the liver of the tumour-bearing host.

As metabolic disturbance is one of the critical features during tumour development, the present work reported the liver metabolic disruption due to the cachectic state under different life stages, which may provide some points to better understand how the liver could be altered in the face of these adverse tumour effects. Indeed, some study limitations should be stated. Our results are restricted to the Walker-256 tumour preclinical model, but more evidence about liver metabolism disturbance in cachexia condition arises. Also, regarding the animal age choice in preclinical studies, young hosts are commonly used in cancer cachexia studies. As we could see, more pronounced cachexia effects were presented in weanling rats, although this condition is not found in the clinic. Therefore, more studies are needed to better understand the liver function in cancer cachexia and guide possible translational processes that improve conventional clinical treatment.

## 4. Materials and Methods

### 4.1. Animals and Experimental Protocol

Weanling (W ≈ 21 days old) and young adult Wistar rats (A, ≈90 days old) were classified according to the developmental stage [49]. The animals obtained from the Animal Facilities at the State University of Campinas, UNICAMP, Brazil) were housed in collective cages under controlled environmental conditions (light and dark 12/12 h; temperature 22 ± 2 °C; and humidity 50–60%). They were monitored daily, weighed 3 times/week and given food and water ad libitum.

The general guidelines of the United Kingdom Co-ordinating Committee on Cancer Research [50] regarding animal welfare were followed, and the Institutional Committee approved the experimental protocol for Ethics in Animal Research (CEEA/IB/UNICAMP, protocol #4918-1/2018; # 5178-1/2019).

W and A animals were randomly distributed into four experimental groups. For tumour-bearing groups, WW and AW rats were implanted with Walker 256 tumour cells (2 × 106 viable cells Walker 256 tumour cells injected subcutaneously into the right flank) and euthanised at pre-agonic state: 14th day of tumour evolution for WW, and 21st day for AW. The control groups (WC and AC) received the same volume injection (0.5 mL) of saline solution (0.9%) subcutaneously into the right flank. The maximal *n* for each group was as follows: WC, *n* = 10; WW, *n* = 12; AC, *n* = 8; AW, *n* = 9. The control groups were euthanised at the same time as the respective tumour groups. After euthanasia, carcass, gastrocnemius muscle, liver and tumour were removed and weighed. Blood samples were centrifuged at 12,000× *g* for 15 min, and the serum was collected and stored at −20 °C. Fragments of liver were frozen directly in liquid nitrogen and stored at −80 °C for further glycogen content, metabolomics, gene and protein expression analyses.

### 4.2. Serum Parameters and Cachexia Indexes

Serum glucose, total serum proteins and albumin were quantified spectrophotometrically using commercial kits (Bioclin, Belo Horizonte, Brazil), according to the manufacturer’s instructions.

The following formula determined cachexia indexes: cachexia index = [(initial body mass—carcass weight + tumour weight + body weight gain of control group)/(initial body mass + body weight gain of control group)] × 100% [51]. The control groups considered in the cachexia index formula were young adult (AC) and weanling (WC), for AW and WW, respectively.

### 4.3. Liver Glycogen and Lipid Quantification

The liver tissue (≈200 mg) was digested with 1 mL of KOH (30%, *w/v*) and heated in a boiling water bath for 15 min. Total glycogen was extracted with ethanol (96%, *v/v*), spun down for 10 min and the final volume was measured. The suspension of total glycogen was added to 20 µL of H_2_SO_4_ and 200 µL of NaOH 1 M and boiled in a water bath for 10 min. The separated fraction was added to glacial acetic acid (94%, *v/v*), ortho-toluidine (6% *v/v*) and thiourea following 10 min of boiling in a water bath and cooled in ice. The total glucose detected was read in a spectrophotometer at 620 nm [52]. Total liver glycogen was expressed as mg/100 g of liver tissue.

Total liver fat was extracted and quantified with petroleum ether using a soxhlet apparatus. One gram of liver tissue was placed in the apparatus, and solvent was added under controlled heating with an electric heating plate. After boiling, rinsing, solvent recovery and pre-drying, the total fat amount was calculated and expressed as a percentage of fat (g per 100 g hepatic tissue), as previously described [53].

### 4.4. Metabolomic Analysis

Liver samples were processed following the protocol established by Le Belle et al. [54]. Briefly, liver tissue fragments were weighed, added to a cold methanol/chloroform solution (2:1 *v/v*, total of 2.5 mL) and sonicated (VCX 500, Vibra-Cell, Sonics & Material Inc., USA) for 3 min with a 10 s pause interval between each minute. Afterward, a cold chloroform/distilled water solution (1:1 *v/v*, total of 2.5 mL) was added to the samples. Samples were briefly vortexed (to form an emulsion) and centrifuged at 3000× *g* for 20 min at 4 °C. The upper phase (containing methanol, water and polar metabolites) was collected and dried in a vacuum concentrator (miVac Duo Concentrator, GeneVac, UK). The remaining solid phase was rehydrated in 0.6 mL of D_2_O-containing phosphate buffer (0.1 M, pH 7.4) and 0.5 mM of TMSP-d4. Samples were added to a 5-mm NMR tube for immediate acquisition.

1D proton nuclear magnetic resonance (^1^H-NMR) spectra acquisition was performed using the Inova Agilent NMR spectrometer (Agilent Technologies Inc., Santa Clara, CA, USA) operating at a frequency of 600 MHz, equipped with a triple resonance cold probe at a constant temperature of 298 K (25 °C). A total of 256 scans were collected with 32-k data points over a spectral width of 8000 Hz. An acquisition time of 4 s and 1.5 s relaxation delay was incorporated between scans, during which a continual water pre-saturation radiofrequency field was applied. After data acquisition, spectroscopic data pre-processing was performed. Manual spectral processing, which included Fourier transform, phasing correction, baseline correction, water region deletion, shim correction, apodisation (line broadening with lb~0.3) and referencing control were applied before the profiling was performed. The identification and quantification of the metabolites were made by computer-assisted manual fitting using the Chenomx RMN Suite software (Chenomx Inc., Edmonton, AB, Canada). To avoid bias, samples were randomly profiled blindly to the evaluator, and identified metabolites fit each spectrum and double-checked by another evaluator. Also, the metabolites found were crosschecked consulting the Chenomx and The Human Metabolome Database (http://hmdb.ca (first accessed on 1 February 2021). The resulting sample profiles consisting of each metabolite and its concentration were presented in millimoles per litre/mg of liver tissue and analysed.

### 4.5. Protein Expression

Samples of liver tissue were lysed in RIPA buffer (150 mM NaCl, 25 mM Tris-Cl, pH 7.4, 5 mM EDTA, 1% (*v/v*) Triton-X, 0.5% (*w/v*) sodium deoxycholate) and supplemented with protease and phosphatase inhibitors. After protein extraction protocol, protein concentration was measured by the bicinchoninic acid (BCA) method, following the manufacturer’s instructions (Pierce™ BCA Protein Assay Kit, Sigma Aldrich, Poole, UK). The proteins (40 μg) were separated by electrophoresis and transferred to nitrocellulose membranes, certifying the transference by staining the proteins with Ponceau S. After that, the membranes were incubated with primary antibodies against PGC1-α (CellSignaling, Danvers, MA, USA, 1:500), mTOR (CellSignaling 1:1000), p mTOR (CellSignalling 1:1000), AMPK (CellSignaling 1:1000), p AMPK (CellSignalling 1:1000) FOXO 1 (CellSignaling 1:1000), citrate synthase (Santa Cruz Biotechnology 1:1000), LDHa (CellSignaling 1:1000), GADPH (Santa Cruz Biotechnology 1:1000, Santa Cruz, CA, USA), OXPHOS (Abcam 1:2000, Cambridge, MA, USA) and vinculin (CellSignaling 1:1000) as a loading control. After that, the membranes were probed with secondary antibodies conjugated with peroxidase, and bands were visualised using a chemiluminescent reagent (ThermoFisher Scientific, Waltham, MA, USA). The membrane images were captured using an image system (Amersham Imager 600, GE Healthcare, Uppsala, Sweden), and band volume quantitation was quantified by Gel-Pro Analyzer software (Media Cibernetics, Inc., Rockville, MD, USA).

### 4.6. Gene Expression

Total RNA from liver tissue was extracted with TRIZOL^®^ reagent (Invitrogen) following the manufacturer’s instructions. The quality of the RNA samples was examined at 260/280 nm and 260/230 nm with a UV spectrophotometer (Nanovue Spectrophotometer 28923215 Ge BioSciences, USA). cDNA was produced using a high-capacity cDNA reverse transcription kit (Applied Biosystems^®^, Waltham, MA, USA) containing Multiscribe^TM^ Reverse Transcriptase. cDNA was synthesised using 1 μg of RNA at 42 °C, according to a high-capacity kit, following the manufacturer’s instructions. Real-time polymerase chain reaction (rt-PCR) was performed using standard methods (ABI Prism 7500 Sequence Detection System; Applied Biosystems, Foster City, CA, USA). Gene expression was normalised considering the expression of the beta-actin gene and calculated by applying the arithmetic formula 2-ΔΔCt. The genes and primer sequences evaluated using rt-PCR are presented in Table S5.

### 4.7. Statistical Analysis

The data analyses were performed by two-way ANOVA, followed by Bonferroni post-hoc test to correct multiple comparisons. We direct the discussion with the following comparisons: WC vs. WW, to extract the tumour effects in the weanling animals; AC vs. AW, to extract the tumour effects in the young adult animals; and WW vs. AW, to extract the age-related differences in the tumour-bearing animals. The student’s *t*-test was applied to comparisons between WW vs. AW for cachexia indexes and relative tumour weight. Data are expressed as means ± standard deviation (SD) and considered significant when *p* ≤ 0.05 (Graph Pad Prism software, version 5.0, San Diego, CA, USA).

## Data Availability

The data presented in this study are available in the article and Supplementary Materials.

## Supplementary Materials

The following are available online at https://www.mdpi.com/article/10.3390/metabo11120831/s1, Figure S1: Representative liver 1H NMR spectra of a control group, Table S1: Morphometric parameters in weanling and adult control and tumour-bearing groups, Table S2: Liver metabolite concentrations in weanling and adult control and tumour-bearing groups, Table S3: Protein expression in the liver tissue in weanling and adult control and tumour-bearing groups, Table S4: Gene expression in the liver tissue in weanling and adult control and tumour-bearing groups, Table S5: Primer sequences for gene expression in the liver tissue in weanling and adult control and tumour-bearing groups.
