# Supplementary material for: Walker-256 Tumour-Induced Cachexia Altered Liver Metabolomic Profile and Function in Weanling and Adult Rats
Source: Metabolites. 2021 Dec 1;11(12):831. doi: 10.3390/metabo11120831 (PMC8705353; doi:10.3390/metabo11120831)
Supplement: Supplementary file 1 [file metabolites-11-00831-s001.zip › FV Supplementary Tables and Figures Miyagut et al Wlaker-256 tumour.pdf]

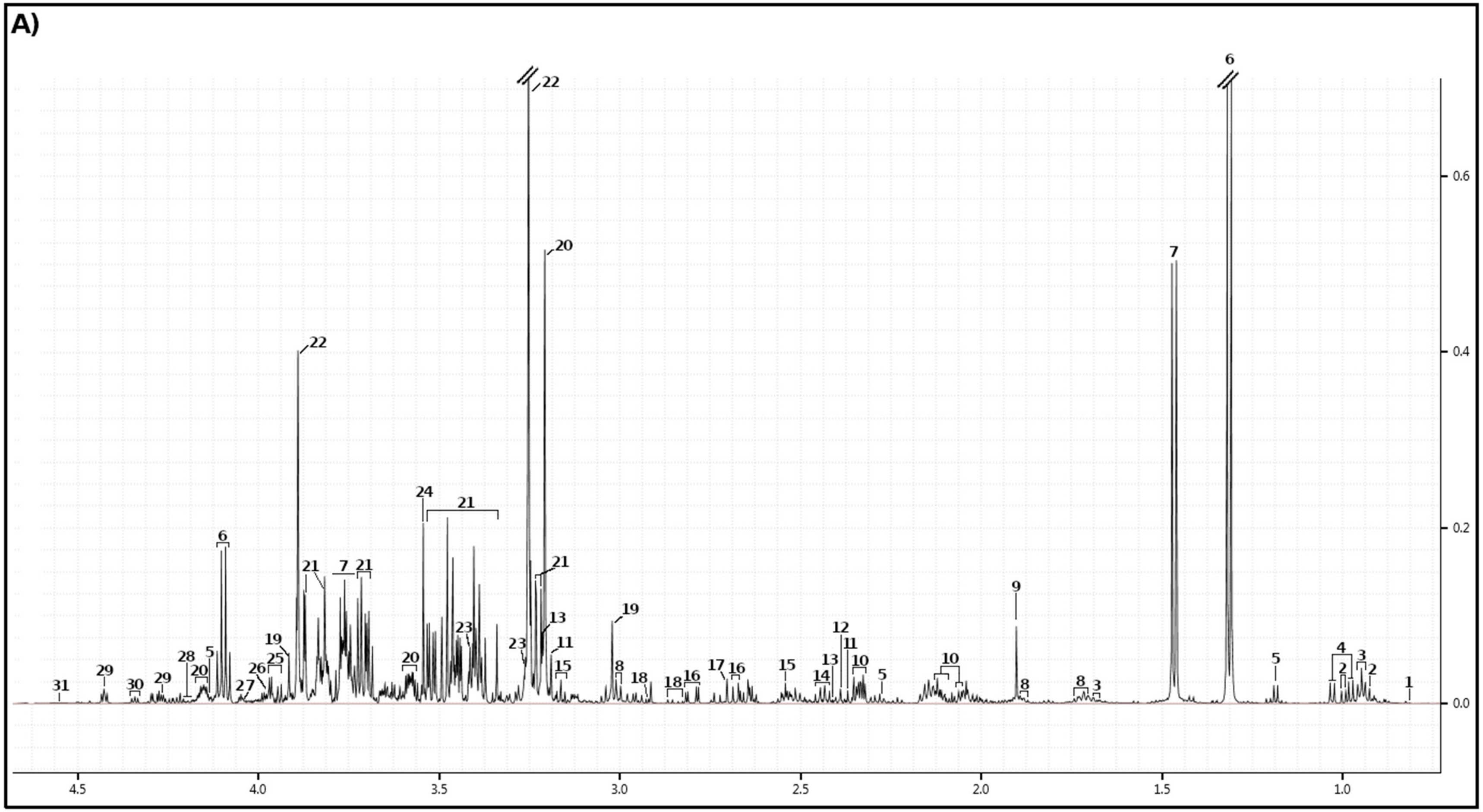


**Figure S1**. *Cont.*

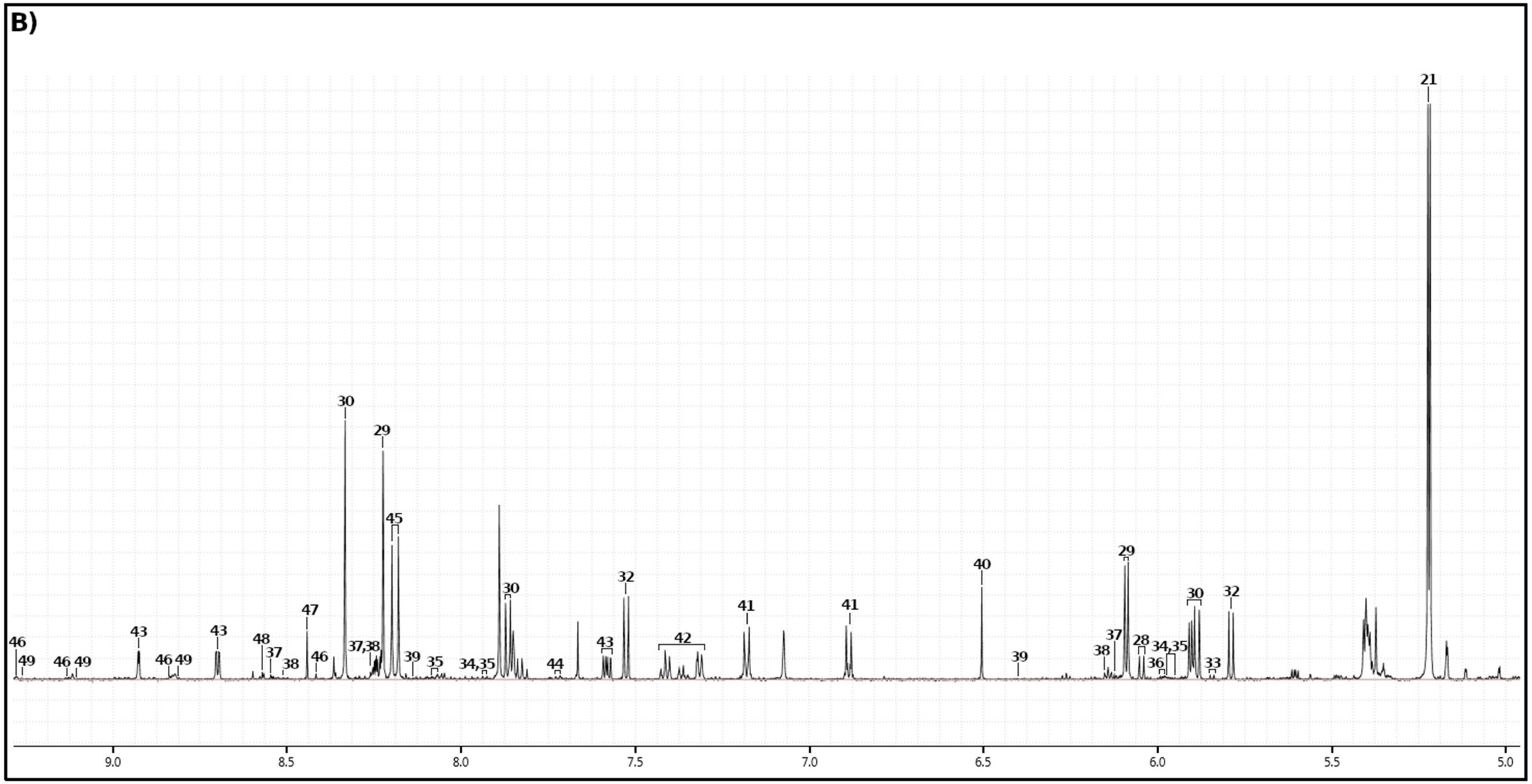


**Figure S1**. Representative liver 1H NMR spectra of a control group. Amplified spectral regions from (**A**) 0-5 ppm (**B**) 5-9.5 ppm. The numbered peaks assigned were: (1) glycocholate; (2) isoleucine; (3) leucine; (4) valine; (5) 3-hydroxybutyrate; (6) lactate; (7) alanine; (8) lysine; (9) acetate; (10) glutamate; (11) pyruvate; (12) succinate; (13) carnitine; (14) glutamine; (15) beta-alanine; (16) aspartate; (17) dimethylamine; (18) asparagine; (19) creatine; (20) O-phosphocholine; (21) glucose; (22) betaine; (23) taurine; (24) glycine; (25) serine; (26) O-phosphoethanolamine; (27) choline; (28) cytidine; (29) inosine; (30) uridine; (31) glutathione; (32) uracil; (33) xanthosine; (34) UDP-N-Acetylglucosamine; (35) UDP-glucose; (36) UMP; (37) AMP; (38) ADP/ATP; (39) GTP; (40) fumarate; (41) tyrosine; (42) phenylalanine; (43) nicotinurate; (44) tryptophan; (45) hypoxanthine; (46) NAD+; (47) formate; (48) IMP; (49) NADP+.

**Table S1**. Morphometric parameters in wealing and adult control and tumour-bearing groups.

| Morphometric and serological parameters | Weanling | | Adult | | F (DFn, DFd) | | | *p*-value | | |
|---|---|---|---|---|---|---|---|---|---|---|
| | WC | WW | AC | AW | | | | | | |
| | Mean ± SD | Mean ± SD | Mean ± SD | Mean ± SD | Interaction | Tumour | Age | Interaction | Tumour | Age |
| Initial weight (g) | 76.00 ± 8.67 | 72.83 ± 9.75 | 273.70 ± 11.58 a | 261.10 ± 8.75 a,c | 1.2 (1.17) | 3.36 (1.17) | 2023 (1.17) | ns | ns | <0.0001 |
| Carcass weight (g) | 115.50 ± 7.84 | 79.33 ± 11.88 a | 242.10 ± 11.89 a,c | 210.80 ± 17.83 a,b,c | 0.17 (1.17) | 32.21 (1.17) | 471.00 (1.17) | ns | < 0.0001 | < 0.0001 |
| Gastrocnemius muscle relative weight (g/g of initial body weight) | 0.012 ± 0.003 | 0.007 ± 0.001 a | 0.006 ± 0.003 a | 0.005 ± 0.001 a | 5.60 (1.17) | 14.52 (1.17) | 31.47 (1.17) | 0.0301 | 0.0014 | < 0.0001 |
| Liver relative weight (g/g of initial body weight) | 0.098 ± 0.014 | 0.083 ± 0.005 | 0.041 ± 0.004 a,c | 0.051 ± 0.001 a,c | 6.54 (1.15) | 0.16 (1.15) | 80.37 (1.15) | ns | ns | < 0.0001 |
| Tumour relative weight (g/g of initial body weight) | - | 0.197 ± 0.048 | - | 0.143 ± 0.031 | - | - | - | - | - | - |
| Cachexia indexes (%) | - | 50.720 ± 5.491 | - | 21.390 ± 6.007 c | - | - | - | - | - | - |
| Glucose (mg/dL) | 115.00 ± 17.28 | 58.93 ± 20.99 a | 127.10 ± 11.64 c | 89.24 ± 8.60 a,b,c | 3.05 (1.35) | 80.96 (1.35) | 16.54 (1.35) | ns | < 0.0001 | 0.0003 |
| Total protein (g/dL) | 5.22 ± 0.28 | 3.88 ± 0.25 a | 5.79 ± 0.48 a,c | 5.12 ± 0.36 b,c | 9.32 (1.35) | 82.84 (1.35) | 67.45 (1.35) | 0.0043 | < 0.0001 | < 0.0001 |
| Albumin (mg/dL) | 3.79 ± 0.49 | 2.79 ± 0.40 a | 4.03 ± 0.23 c | 3.28 ± 0.33 b,c | 1.01 (1.35) | 49.00 (1.35) | 8.36 (1.35) | ns | < 0.0001 | 0.0065 |

**Legend:** (WC) weanling control group (n=10); (WW) weanling tumour-bearing group (n=12); (AC) adult control group (n=8); (AW) adult tumour-bearing group (n=9). The relative weight values were obtained by dividing the absolute organ weight by each animal's respective initial body weight (expressed as g/g of initial weight). Cachexia index = [(initial body mass – carcass weight + tumour weight + body weight gain of control group)/ (initial body mass + body weight gain of control group)] x 100% (expressed as %). Data were expressed as mean ± standard deviation (SD). For the relative weights and serum dosages, data analyses were accessed by two-way ANOVA and corrected for multiple comparisons by the post hoc test Bonferroni. For tumour relative weight and cachexia indexes, a student´s t test was performed. DFn and DFd: degrees of freedom for the numerator and denominator of the F ratio, respectively. Differences were significant when $p \leq 0.05$. a represents differences from the WC group; b indicates differences from AC group, and c indicates differences from the WW group.

**Table S2**. Liver metabolites concentration in weanling and adult control and tumour-bearing groups.

| Metabolites | Weanling | | Adult | | F (DFn, DFd) | | | *p*-value | | |
|---|---|---|---|---|---|---|---|---|---|---|
| | WC | WW | AC | AW | | | | | | |
| | Mean ± SD | Mean ± SD | Mean ± SD | Mean ± SD | Interaction | Tumour | Age | Interaction | Tumour | Age |
| 3-Hydroxybutyrate | 24.19 ± 11.63 | 50.63 ± 15.72 a | 29.51 ± 10.73 | 40.28 ± 21.27 | 1.74 (1.25) | 9.85 (1.25) | 0.18 (1.25) | ns | 0.0043 | ns |
| ADP/ATP | 7.94 ± 3.87 | 12.53 ± 9.46 | 11.51 ± 6.76 | 15.76 ± 4.23 | 0.09 (1.27) | 0.09 (1.27) | 0.18 (1.27) | ns | ns | ns |
| AMP | 5.62 ± 3.39 | 9.05 ± 5.78 | 12.02 ± 7.03 | 11.52 ± 5.81 | 0.92 (1.27) | 0.51 (1.27) | 4.67 (1.27) | ns | ns | 0.0398 |
| Acetate | 66.94 ± 39.99 | 133.00 ± 62.22 | 100.70 ± 62.70 | 148.3 ± 72.61 | 0.17 (1.27) | 6.46 (1.27) | 1.21 (1.27) | ns | 0.0171 | ns |
| Alanine | 454.20 ± 361.90 | 678.80 ± 281.60 | 265.70 ± 162.40 c | 513.10 ± 196.30 | 0.01 (1.27) | 5.92 (1.27) | 3.33 (1.27) | ns | 0.0218 | ns |
| Asparagine | 24.06 ± 17.72 | 40.67 ± 16.18 | 12.96 ± 11.50 c | 34.55 ± 22.81 | 0.15 (1.27) | 8.63 (1.27) | 1.75 (1.27) | ns | 0.0067 | ns |
| Aspartate | 83.13 ± 66.26 | 188.30 ± 56.36 a | 117.50 ± 56.37 | 145.10 ± 68.02 | 2.95 (1.27) | 8.63 (1.27) | 0.04 (1.27) | ns | 0.0067 | ns |
| Betaine | 392.00 ± 269.60 | 925.60 ± 319.90 a | 192.60 ± 218.60 c | 416.50 ± 162.50 c | 2.72 (1.27) | 16.28 (1.27) | 14.24 (1.27) | ns | 0.0004 | 0.0008 |
| Carnitine | 33.49 ± 17.31 | 49.96 ± 36.75 | 25.07 ± 13.37 | 67.13 ± 33.49 | 1.46 (1.27) | 7.62 (1.27) | 0.17 (1.27) | ns | 0.0102 | ns |
| Choline | 15.72 ± 7.69 | 36.12 ± 9.45 a | 12.00 ± 4.50 c | 44.64 ± 19.41 a,b | 1.64 (1.24) | 30.80 (1.24) | 0.25 (1.24) | ns | <0.0001 | ns |
| Creatine | 35.68 ± 33.30 | 121.90 ± 82.36 a | 26.23 ± 20.68 c | 49.56 ± 24.08 c | 2.70 (1.27) | 8.20 (1.27) | 4.57 (1.27) | ns | 0.0080 | 0.0417 |
| Cytidine | 8.09 ± 7.38 | 16.68 ± 8.45 | 3.61 ± 0.52 c | 12.72 ± 5.85 | 0.01 (1.26) | 11.86 (1.26) | 2.69 (1.26) | ns | 0.0020 | ns |
| Dimethylamine | 10.97 ± 4.07 | 13.11 ± 9.81 | 3.79 ± 4.26 | 16.20 ± 6.04 b | 4.06 (1.27) | 8.15 (1.27) | 0.64 (1.27) | ns | 0.0082 | ns |

| | | | | | | | | | | |
|---|---|---|---|---|---|---|---|---|---|---|
| Formate | 18.78 ± 8.71 | 23.65 ± 4.69 | 17.08 ± 5.03 | 28.21 ± 11.48 | 1.11 (1.26) | 7.27 (1.26) | 0.23 (1.26) | ns | 0.0121 | ns |
| Fumarate | 11.56 ± 7.54 | 19.39 ± 5.45 | 7.78 ± 7.04 c | 16.22 ± 9.77 | 0.01 (1.27) | 8.77 (1.27) | 1.60 (1.27) | ns | 0.0063 | ns |
| GTP | 5.41 ± 2.51 | 11.04 ±11.13 | 16.77 ± 8.28 | 15.39 ± 7.89 | 1.29 (1.27) | 0.47 (1.27) | 6.46 (1.27) | ns | ns | 0.0171 |
| Glucose | 2157 ± 718.30 | 2428 ± 1179 | 2464 ± 1085 | 3578 ± 1046 | 1.23 (1.27) | 3.32 (1.27) | 3.38 (1.27) | ns | ns | ns |
| Glutamate | 210.00 ± 95.03 | 343.2 ± 73.02 | 214.7 ± 182.60 | 371.9 ± 153.60 | 0.07 (1.27) | 9.81 (1.27) | 0.13 (1.27) | ns | 0.0041 | ns |
| Glutamine | 106.10 ± 43.34 | 212.4 ± 74.03 a | 94.69 ±28.90 c | 204.10 ± 98.04 b | $4x10^{-3}$ (1.27) | 17.82 (1.27) | 0.15 (1.27) | ns | 0.0002 | ns |
| Glutathione | 55.16 ± 47.19 | 36.82 ± 31.55 | 120.2 ± 82.94 | 114.10 ± 86.82 | 0.07 (1.26) | 0.26 (1.26) | 8.90 (1.26) | ns | ns | 0.0061 |
| Glycine | 174.30 ± 85.18 | 337.30 ± 73.37 a | 215. 6 ± 91.38 | 378.8 ± 134.70 a,b | $5x10^{-6}$ (1.27) | 20.61 (1.27) | 1.33 (1.27) | ns | 0.0001 | ns |
| Glycocholate | 2.81 ± 0.89 | 10.20 ± 3.87 a | 9.37 ± 7.51 | 12.30 ± 4.58 a | 1.72 (1.26) | 9.22 (1.26) | 6.48 (1.26) | ns | 0.0054 | 0.0172 |
| Hypoxanthine | 48.83 ± 33.52 | 97.90 ± 38.62 c | 22.64 ± 7.83 | 105.00 ± 63.87 b | 1.04 (1.26) | 16.15 (1.26) | 0.34 (1.26) | ns | 0.0004 | ns |
| IMP | 3.58 ± 1.60 | 8.70 ± 5.80 | 6.76 ± 6.28 | 10.19 ± 6.08 | 0.17 (1.26) | 4.42 (1.26) | 1.32 (1.26) | ns | 0.0455 | ns |
| Inosine | 88.33 ± 39.16 | 138.00 ± 31.51 | 66.80 ± 46.46 | 204.60 ± 78.36 a,b | 5.46 (1.27) | 24.70 (1.27) | 1.43 (1.27) | 0.0272 | <0.0001 | ns |
| Isoleucine | 16.84 ± 11.59 | 31.28 ± 2.09 | 15.81 ± 8.62 | 37.90 ± 15.52 a,b | 0.96 (1.26) | 21.94 (1.26) | 0.52 (1.26) | ns | <0.0001 | ns |
| Lactate | 1300 ± 436.30 | 1642 ± 506.10 | 955.20 ± 410.40 | 2100 ± 815.90 b | 3.64 (1.27) | 12.47 (1.27) | 0.07 (1.27) | ns | 0.0015 | ns |
| Leucine | 42.78 ± 21.72 | 66.25 ± 3.46 | 34.14 ± 15.97 | 85.57 ± 36.42 a,b | 2.76 (1.26) | 19.83 (1.26) | 0.40 (1.26) | ns | 0.0001 | ns |

| | | | | | | | | | | |
|---|---|---|---|---|---|---|---|---|---|---|
| Lysine | 58.80 ± 41.56 | 105.20 ± 30.38 | 50.40 ± 35.08 | 108.90 ± 51.21 | 0.17 (1.27) | 12.88 (1.27) | 0.03 (1.27) | ns | 0.0013 | ns |
| NAD+ | 8.54 ± 5.23 | 10.92 ± 3.33 | 27.60 ± 16.20 a,c | 11.18 ± 4.46 b | 8.31 (1.27) | 4.27 (1.27) | 8.83 (1.27) | 0.0076 | 0.0486 | 0.0062 |
| NADP+ | 3.41 ± 1.55 | 3.88 ± 1.85 | 5.27 ± 2.25 | 6.49 ± 2.53 a | 0.24 (1.26) | 1.21 (1.26) | 8.47 (1.26) | ns | ns | 0.0073 |
| Nicotinurate | 41.52 ± 15.06 | 63.61 ± 7.92 | 44.11 ± 21.37 | 81.48 ± 30.81 a,b | 1.09 (1.27) | 16.46 (1.27) | 1.95 (1.27) | ns | 0.0004 | ns |
| O-Phosphocholine | 244.20 ± 75.86 | 291.5 ± 111.10 | 316.30 ± 147.60 | 639.90 ± 241.30 a,b,c | 5.83 (1.27) | 10.49 (1.27) | 13.49 (1.27) | 0.0229 | 0.0032 | 0.0010 |
| O-Phosphoethanolamine | 56.19 ± 36.10 | 154.5 ± 70.86 a | 37.32 ± 11.12 c | 99.02 ± 68.70 | 0.71 (1.26) | 13.55 (1.26) | 2.93 (1.26) | ns | 0.0011 | ns |
| Phenylalanine | 17.73 ± 9.01 | 28.16 ± 2.58 | 14.20 ± 8.01 | 33.33 ± 14.38 a,b | 1.56 (1.26) | 18.04 (1.26) | 0.06 (1.26) | ns | 0.0002 | ns |
| Pyruvate | 9.26 ± 5.36 | 16.36 ± 5.67 | 8.69 ± 6.66 | 16.84 ± 9.92 | 0.04 (1.27) | 8.56 (1.27) | $2x10^{-4}$ (1.27) | ns | 0.0069 | ns |
| Serine | 80.81 ± 43.94 | 148.00 ± 50.18 | 117.4 ± 74.43 | 269.5 ± 175.50 a | 1.32 (1.27) | 8.84 (1.27) | 4.59 (1.27) | ns | 0.0061 | 0.0414 |
| Succinate | 77.51 ± 49.94 | 100.6 ± 46.54 | 89.04 ± 35.06 | 101.5 ± 35.88 | 0.11 (1.27) | 1.29 (1.27) | 0.16 (1.27) | ns | ns | ns |
| Taurine | 426.2 ± 358.30 | 426.20 ± 205.80 | 671.60 ± 461.30 | 969.00 ± 568.80 | 0.44 (1.27) | 1.78 (1.27) | 5.34 (1.27) | ns | ns | 0.0287 |
| Tryptophan | 4.53 ± 2.00 | 8.15 ± 1.79 a | 5.47 ± 1.76 | 9.79 ± 3.31 a,b | 0.17 (1.27) | 21.92 (1.27) | 2.32 (1.27) | ns | <0.0001 | ns |
| Tyrosine | 21.91 ± 15.03 | 31.75 ± 3.52 | 15.83 ± 9.18 | 38.63 ± 15.50 a,b | 2.25 (1.26) | 14.27 (1.26) | 0.01 (1.26) | ns | 0.0008 | ns |
| UDP-N-Acetylglucosamine | 6.33 ± 3.94 | 15.72 ± 12.84 | 16.77 ± 7.36 | 21.72 ± 11.39 a | 0.36 (1.27) | 3.77 (1.27) | 4.96 (1.27) | ns | ns | 0.0344 |
| UDP-glucose | 14.48 ± 7.39 | 23.15 ± 19.46 | 25.93 ± 8.57 | 35.67 ± 16.49 | 0.01 (1.27) | 2.85 (1.27) | 4.83 (1.27) | ns | ns | 0.0366 |

| | | | | | | | | | | |
|---|---|---|---|---|---|---|---|---|---|---|
| UMP | 9.76 ± 10.42 | 4.09 ± 1.84 | 4.30 ± 1.88 | 9.39 ± 7.28 | 4.42 (1.24) | 0.01 (1.24) | $9 \times 10^{-4}$ (1.24) | 0.0461 | ns | ns |
| Uracil | 9.33 ± 10.81 | 37.88 ± 27.60 | 1.15 ± 0.64 c | 26.62 ± 20.38 | 0.04 (1.25) | 11.92 (1.25) | 1.54 (1.25) | ns | 0.0020 | ns |
| Uridine | 33.38 ± 21.13 | 40.17 ± 18.07 | 9.57 ± 2.46 | 76.44 ± 31.41 a,b,c | 13.17 (1.26) | 19.80 (1.26) | 0.57 (1.26) | 0.0012 | 0.0001 | ns |
| Valine | 32.94 ± 18.58 | 54.36 ± 3.47 | 26.32 ± 15.05 c | 67.04 ± 27.00 a | 2.11 (1.26) | 21.89 (1.26) | 0.21 (1.26) | ns | <0.0001 | ns |
| Xanthosine | 2.47 ± 1.15 | 2.80 ± 1.37 | 1.98 ± 0.91 | 5.66 ± 3.33 a,b,c | 5.34 (1.27) | 7.60 (1.27) | 2.68 (1.27) | 0.0287 | 0.0104 | ns |
| B-Alanine | 40.27 ± 23.53 | 75.72 ± 27.16 a | 16.60 ± 3.79 c | 44.47 ± 19.33 c | 0.21 (1.26) | 14.54 (1.26) | 10.93 (1.26) | ns | 0.008 | 0.0028 |

**Legend:** WC weanling control group n=7; WW, weanling tumour-bearing group n=10; AC, adult control group n=6; AW, adult tumour-bearing group n=8. Data were expressed as mean ± standard deviation (SD) in millimolar/milligram of liver tissue. Data were analysed by two-way ANOVA and corrected for multiple comparisons using the post hoc test Bonferroni. DFn and DFd: degrees of freedom for the numerator and denominator of the F ratio, respectively. Differences were significant when $p \leq 0.05$. a represents differences from the WC group; b indicates differences from AC group, and c indicates differences from the WW group.

**Table S3**. Protein expression in the liver tissue in weanling and adult control and tumour-bearing groups.

| **Protein** | **Weanling** | | **Adult** | | **F (DFn, DFd)** | | | ***p*-value** | | |
|---|---|---|---|---|---|---|---|---|---|---|
| | **WC** | **WW** | **AC** | **AW** | | | | | | |
| | **Mean ± SD** | **Mean ± SD** | **Mean ± SD** | **Mean ± SD** | **Interaction** | **Tumour** | **Age** | **Interaction** | **Tumour** | **Age** |
| Cytrate synthase | 0.86 ± 0.32 | 1.00 ± 0.19 | 1.12 ± 0.16 | 1.34 ± 0.18 a,c | 0.23 (1.19) | 3.75 (1.19) | 10.93 (1.19) | ns | ns | 0.0037 |
| GADPH | 1.14 ± 0.99 | 0.08 ± 0.09 a | 0.46 ± 0.45 | 0.11 ± 0.07 a | 3.01 (1.20) | 11.8 (1.20) | 2.56 (1.20) | ns | 0.0026 | ns |
| LDHA | 1.07 ± 0.29 | 1.36 ± 0.27 | 1.12 ± 0.26 | 1.17 ± 0.23 | 1.19 (1.20) | 2.38 (1.20) | 0.35 (1.20) | ns | ns | ns |
| mTOR | 0.72 ± 0.40 | 1.28 ± 0.34 | 1.29 ± 0.62 | 2.01 ± 0.77 a | 0.13 (1.20) | 7.30 (1.20) | 7.71 (1.20) | ns | 0.0137 | 0.0116 |
| p mTOR | 0.19 ± 0.20 | 0.62 ± 0.44 | 0.68 ± 0.52 | 1.50 ± 0.72 a,c | 0.76 (1.19) | 7.99 (1.19) | 9.59 (1.19) | ns | 0.0108 | 0.0059 |
| AMPK | 0.60 ± 0.18 | 0.19 ± 0.15 a | 0.28 ± 0.24 | 0.32 ± 0.28 | 5.79 (1.19) | 3.78 (1.19) | 0.99 (1.19) | 0.0264 | ns | ns |
| p AMPK | 0.75 ± 0.31 | 1.05 ± 0.33 | 0.79 ± 0.64 | 0.92 ± 0.59 | 0.20 (1.20) | 1.15 (1.20) | 0.05 (1.20) | ns | ns | ns |
| FOXO1 | 0.61 ± 0.49 | 0.59 ± 0.23 | 0.64 ± 0.01 | 0.41 ± 0.27 | 0.85 (1.18) | 1.27 (1.18) | 0.40 (1.18) | ns | ns | ns |
| PCG1α | 0.95 ± 0.07 | 0.95 ± 0.35 | 1.03 ± 0.30 | 0.83 ± 0.14 | 0.36 (1.18) | 0.41 (1.18) | 0.01 (1.18) | ns | ns | ns |
| Mitochondrial complex I | 0.18 ± 0.16 | 0.36 ± 0.24 | 0.23 ± 0.14 | 0.36 ± 0.27 | 0.06 (1.20) | 2.54 (1.20) | 0.06 (1.20) | ns | ns | ns |
| Mitochondrial complex II | 1.16 ± 0.83 | 0.15 ± 0.25 a | 0.39 ± 0.23 | 0.61 ± 0.51 | 8.62 (1.20) | 3.50 (1.20) | 0.55 (1.20) | 0.0082 | ns | ns |
| Mitochondrial complex III | 0.97 ± 0.43 | 0.88 ± 0.32 | 0.78 ± 0.44 | 1.15 ± 0.56 | 1.49 (1.20) | 0.55 (1.20) | 0.06 (1.20) | ns | ns | ns |
| Mitochondrial complex IV | 0.60 ± 0.15 | 0.15 ± 0.17 a | 0.24 ± 0.05 | 0.46 ± 0.25 c | 14.19 (1.18) | 1.71 (1.18) | 0.07 (1.18) | 0.0014 | ns | ns |

| | | | | | | | | | | |
|---|---|---|---|---|---|---|---|---|---|---|
| Mitochondrial complex V | 1.17 ± 0.29 | 1.21 ± 0.24 | 0.94 ± 0.23 | 1.09 ± 0.26 | 0.26 (1.19) | 0.75 (1.19) | 2.46 (1.19) | ns | ns | ns |

**Legend:** WC, weanling control group n=5; WW, weanling tumour-bearing group n=8; AC, adult control group n=4; AW, adult tumour-bearing group n=7. Data were expressed as mean ± standard deviation (SD). The units used were protein/ vinculin protein expression (optical density, OD - western blot image). Data were analysed by two-way ANOVA and corrected for multiple comparisons using the post hoc test Bonferroni. DFn and DFd: degrees of freedom for the numerator and denominator of the F ratio, respectively. Differences were significant when $p \leq 0.05$. a represents differences from the WC group; b indicates differences from AC group, and c indicates differences from the WW group.

**Table S4**. Gene expression in the liver tissue in weanling and adult control and tumour-bearing groups.

| Gene | Weanling | | Adult | | F (DFn, DFd) | | | *p*-value | | |
|---|---|---|---|---|---|---|---|---|---|---|
| | WC | WW | AC | AW | | | | | | |
| | **Mean ± SD** | **Mean ± SD** | **Mean ± SD** | **Mean ± SD** | **Interaction** | **Tumour** | **Age** | **Interaction** | **Tumour** | **Age** |
| *CREB1* | 0.85 ± 0.29 | 0.35 ± 0.08 | 1.07 ± 0.42 c | 1.09 ± 0.23 c | 4.96 (1.17) | 3.97 (1.17) | 16.51 (1.17) | 0.0397 | ns | 0.0008 |
| *PEPCK* | 1.38 ± 0.90 | 13.79 ± 9.57 a | 1.22 ± 0.79 | 1.00 ± 0.48 c | 8.98 (1.19) | 8.37 (1.19) | 9.45 (1.19) | 0.0074 | 0.0093 | 0.0063 |
| *PPAR α* | 1.15 ± 0.62 | 1.58 ± 0.97 | 1.15 ± 0.66 | 0.51 ± 0.17 c | 3.78 (1.19) | 0.14 (1.19) | 3.67 (1.19) | ns | ns | ns |
| *AMPK* | 1.26 ± 0.72 | 0.76 ± 0.33 | 0.75 ± 0.31 | 0.68 ± 0.28 | 1.26 (1.19) | 2.13 (1.19) | 2.30 (1.19) | ns | ns | ns |
| *GS3K* | 1.25 ± 0.79 | 0.66 ± 0.49 | 1.10 ± 0.55 | 0.89 ± 0.23 | 0.65 (1.19) | 3.00 (1.19) | 0.03 (1.19) | ns | ns | ns |
| *CRP* | 0.87 ± 0.48 | 0.27 ± 0.11 | 1.07 ± 0.42 c | 0.68 ± 0.33 | 0.44 (1.17) | 9.97 (1.17) | 3.76 (1.17) | ns | 0.0058 | ns |
| *mTOR* | 0.86 ± 0.27 | 0.44 ± 0.33 | 1.08 ± 0.42 c | 0.93 ± 0.29 | 0.93 (1.18) | 4.06 | 6.43 | ns | ns | 0.0207 |
| *PGC1 α* | 1.51 ± 1.37 | 1.59 ± 0.88 | 1.28 ± 0.87 | 0.76 ± 0.12 | 0.56 (1.18) | 0.31 (1.18) | 1.80 (1.18) | ns | ns | ns |
| *GAPDH* | 0.82 ± 0.15 | 0.41 ± 0.15 | 1.11 ± 0.15 | 1.02 ± 0.52 | 0.81 (1.17) | 1.96 (1.17) | 6.11 (1.17) | ns | ns | 0.0244 |
| *FOXO 1* | 1.26 ± 0.85 | 0.79 ± 0.66 | 1.13 ± 0.57 | 0.71 ± 0.18 | 0.01 (1.19) | 3.01 (1.19) | 0.16 (1.19) | ns | ns | ns |

**Legend:** WC, weanling control group n=5; WW, weanling tumour-bearing group n=8; AC, adult control group n=4; AW, adult tumour-bearing group n=7. Data were expressed as mean ± standard deviation (SD). The units used were relative protein expression (rtPCR, expressed as arbitrary units - AU). Data were analysed by two-way ANOVA and corrected for multiple comparisons using the post hoc test Bonferroni. DFn and DFd: degrees of freedom for the numerator and denominator of the F ratio, respectively. Differences were significant when $p \leq 0.05$. a represents differences from the WC group; b indicates differences from AC group, and c indicates differences from the WW group.

**Table S5.** Primer sequences for gene expression in the liver tissue in weanling and adult control and tumour-bearing groups.

| **Genes** | **Primer sequences** |
|---|---|
| *AMPK* | *Forward*: 5′ CAG TTG GAC TAT GAA TGG AAG 3′<br>*Reverse*: 5′ CCT GAT TTG GCT TCT GTA ATC 3′ |
| *PPAR α* | *Forward*: 5′ GCT CTG AAC CAT TGG CGT TCG 3′<br>*Reverse*: 5′ TGT CAG TTC ACA GGG AAG GC 3′ |
| *CRP* | *Forward*: 5′ ATC ACG ATA AGC TTC TCT CAG GC 3′<br>*Reverse*: 5′ ACT CCG GGA AAT ACG AAG GC 3′ |
| *CREB1* | *Forward*: 5′ GAC CAT GGA CTC TGG AGC AG 3′<br>*Reverse*: 5′ TGA GCT GCT GGC ATG GAT AC 3′ |
| *FOXO 1* | *Forward*: 5′ CGG AGA TAC CTT GGA TTT TAA C 3′<br>*Reverse*: 5′ TTT AAA TGT TGC CTG CTC AC 3′ |
| *GS3K* | *Forward*: 5′ TTT TGA TGA ATT ACG GGA CC 3′<br>*Reverse*: 5′ GTT ACT TGA CAG TTC TTG AGT G 3′ |
| *mTOR* | *Forward*: 5′ AGA AAT TTG ATC AGG TGT GC 3′<br>*Reverse*: 5′ TTC CTT TTC CTT CTT GAC AC 3′ |
| *PGC1 α* | *Forward*: 5′ CTG GTT GCC TGC ATG AGT GT 3′<br>*Reverse*: 5′ GTT CGC AGG CTC ATT GTT GT 3′ |
| *GAPDH* | *Forward*: 5′ CCA TGG AGA AGG CTG GG 3′<br>*Reverse*: 5′ CAA AGT TGT CAT GGA TGA CC 3′ |
| *PEPCK* | *Forward*: 5′ AAA ACA CCA TCT TCA CCA AC 3′<br>*Reverse*: 5′ AAT AAT GGG ACA TTG GCT G 3′ |
