# Supplementary material for: Walker-256 Tumour-Induced Cachexia Altered Liver Metabolomic Profile and Function in Weanling and Adult Rats
Source: Metabolites. 2021 Dec 1;11(12):831. doi: 10.3390/metabo11120831 (PMC8705353; doi:10.3390/metabo11120831)
Supplement: Supplementary file 1 [file metabolites-11-00831-s001.zip › WB material suplementar 160921.pptx]

## Slide 1
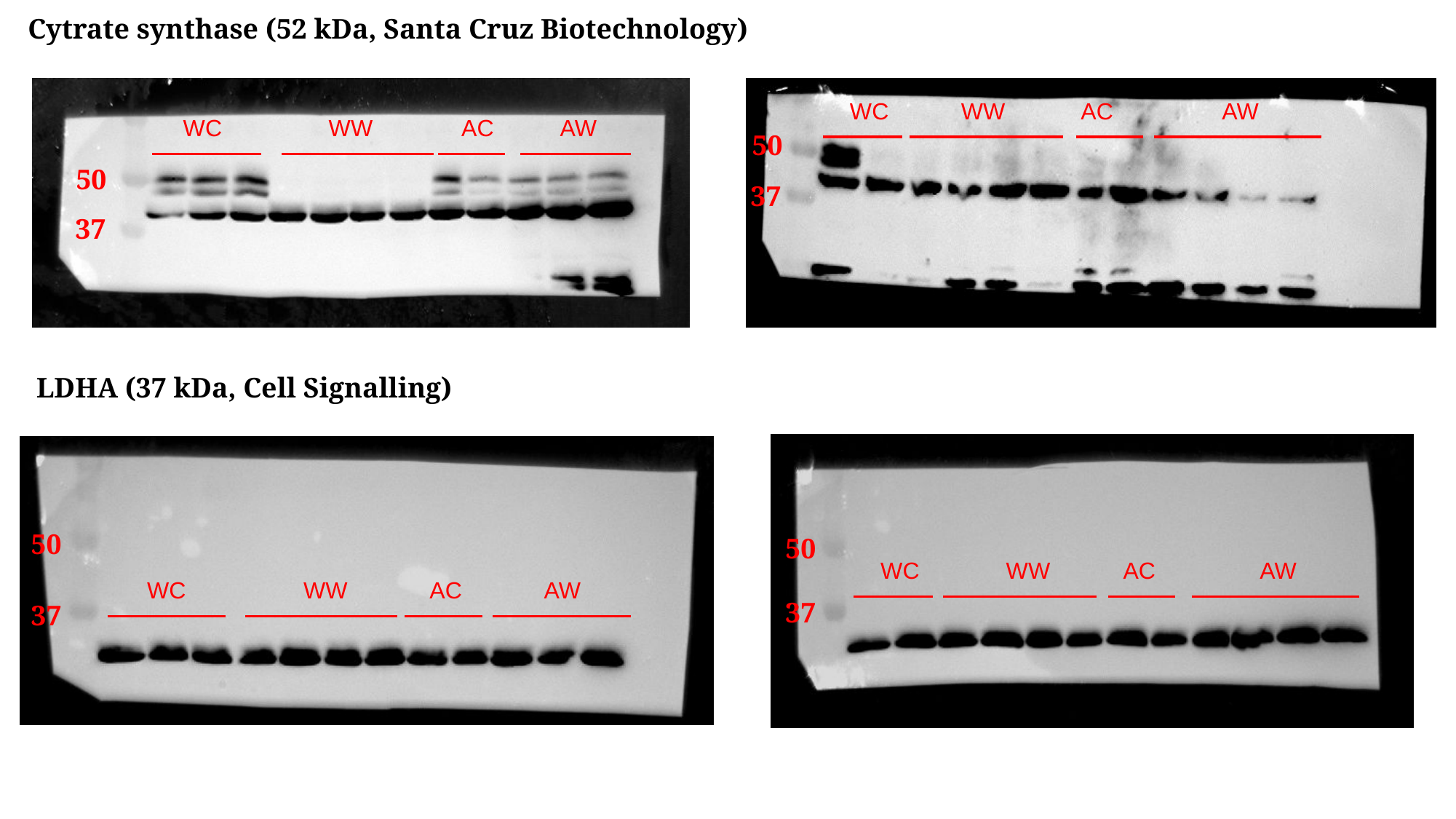

Cytrate synthase (52 kDa, Santa Cruz Biotechnology)
WC
WW
AC
AW
WC
WW
AC
AW
50
50
37
37
LDHA (37 kDa, Cell Signalling)
50
50
WC
WW
AC
AW
WC
WW
AC
AW
37
37

## Slide 2
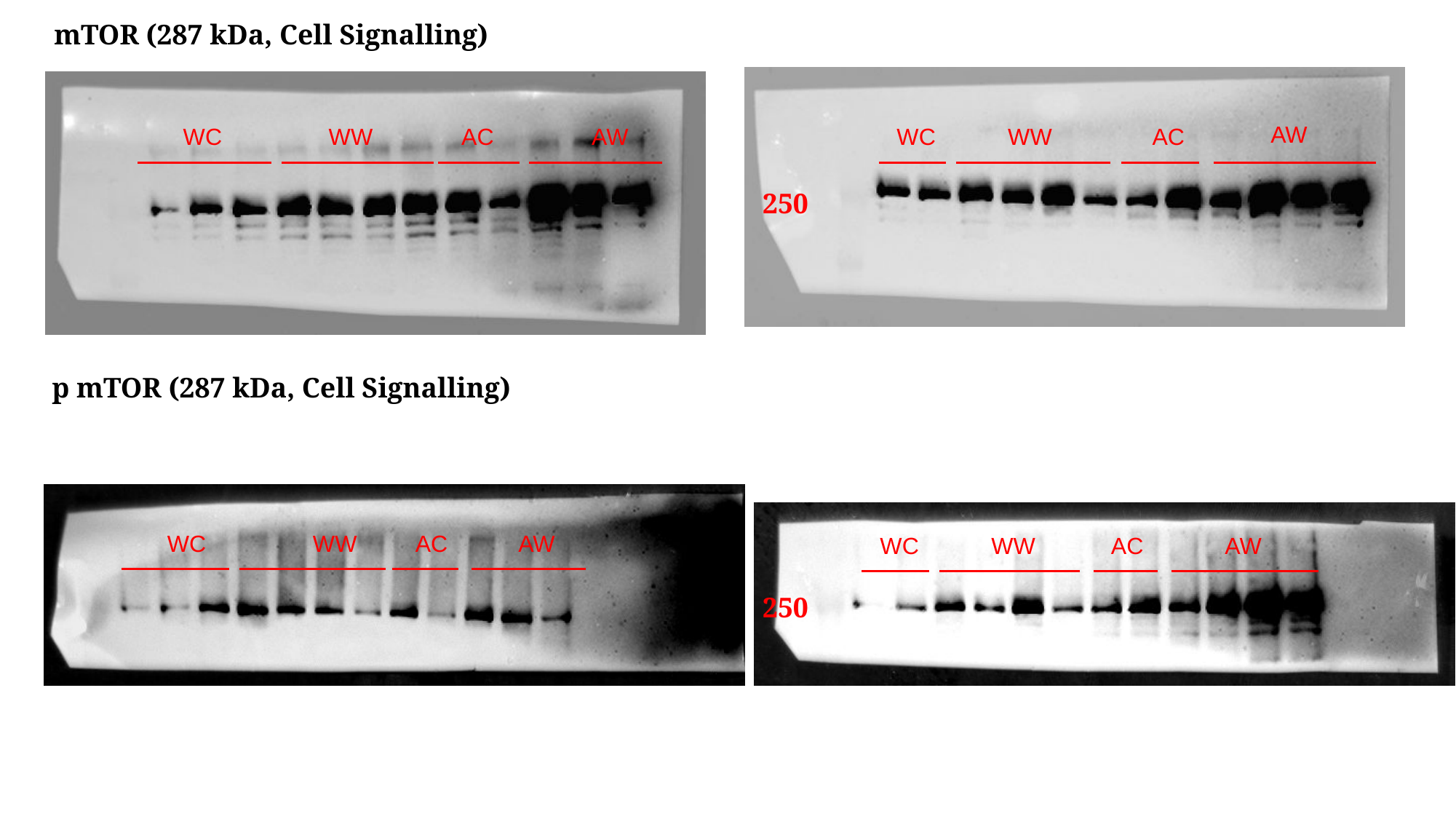

mTOR (287 kDa, Cell Signalling)
AW
WC
WW
AC
AW
WC
WW
AC
250
p mTOR (287 kDa, Cell Signalling)
WC
WW
AC
AW
WC
WW
AC
AW
250

## Slide 3
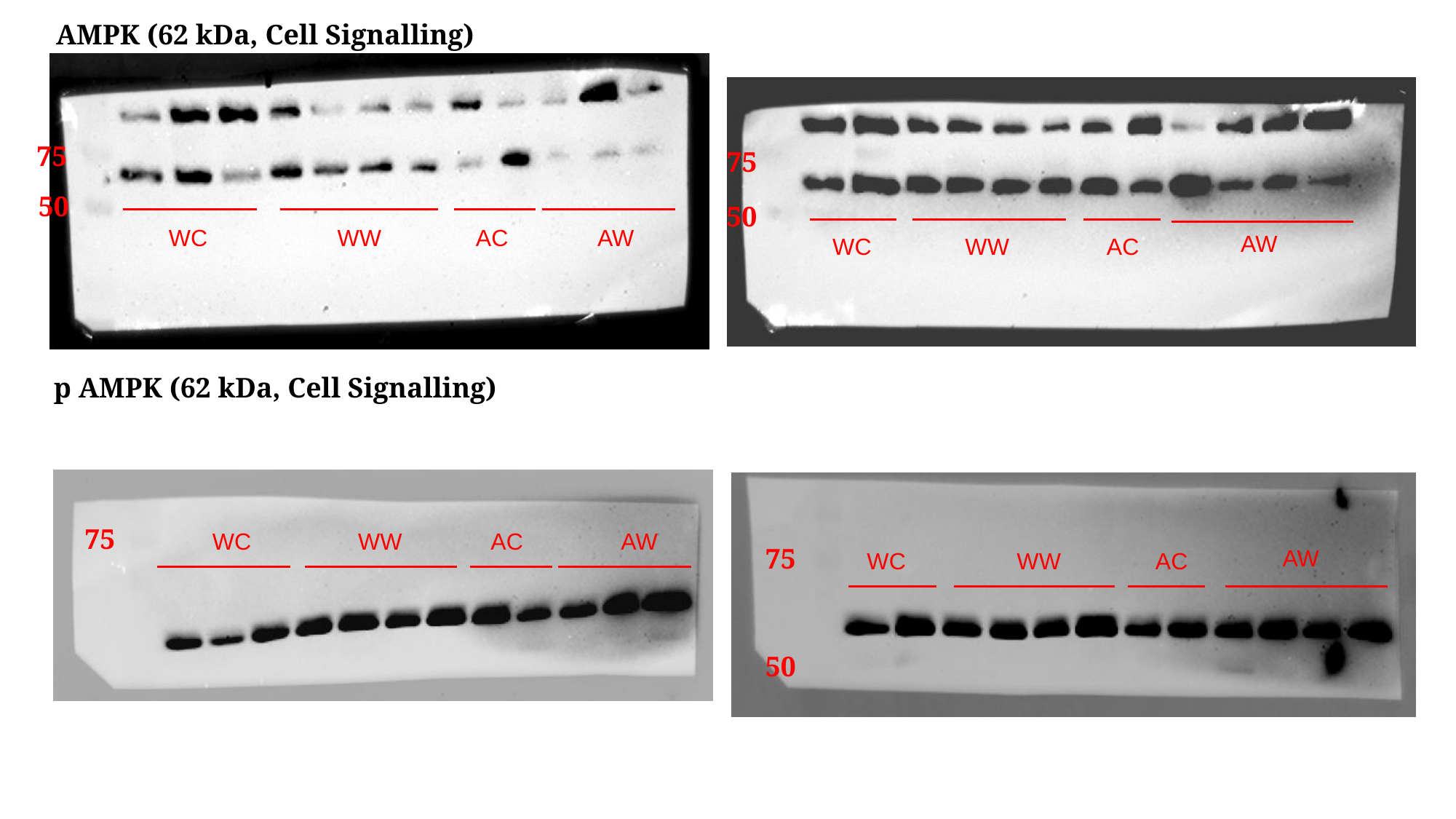

AMPK (62 kDa, Cell Signalling)
75
75
50
50
WC
WW
AC
AW
AW
WC
WW
AC
p AMPK (62 kDa, Cell Signalling)
75
WC
WW
AC
AW
75
AW
WC
WW
AC
50

## Slide 4
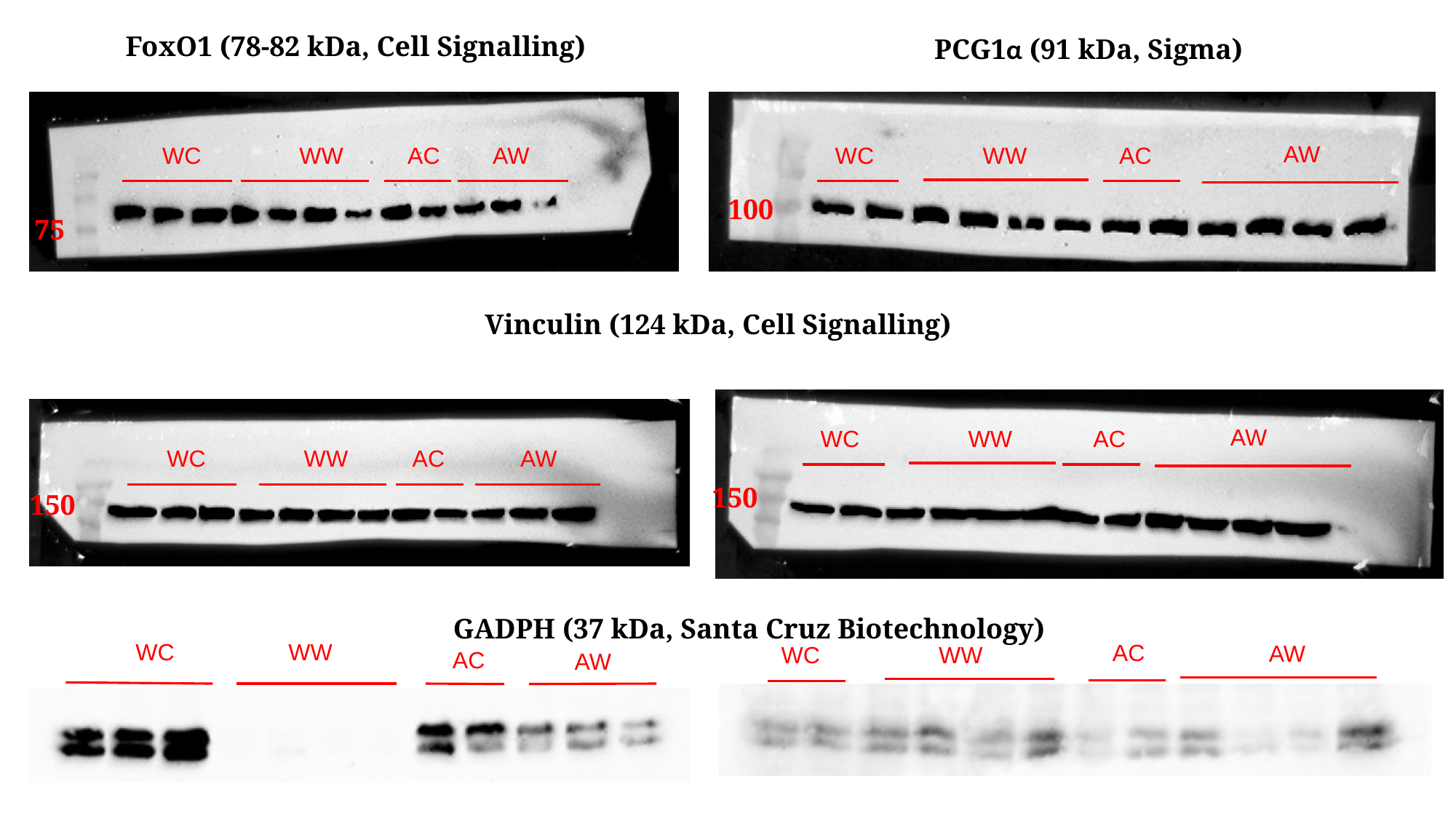

FoxO1 (78-82 kDa, Cell Signalling)
PCG1α (91 kDa, Sigma)
AW
WC
WW
AC
AW
WC
WW
AC
100
75
Vinculin (124 kDa, Cell Signalling)
AW
WC
WW
AC
WC
WW
AC
AW
150
150
GADPH (37 kDa, Santa Cruz Biotechnology)
WC
WW
AC
AW
WC
WW
AC
AW

## Slide 5
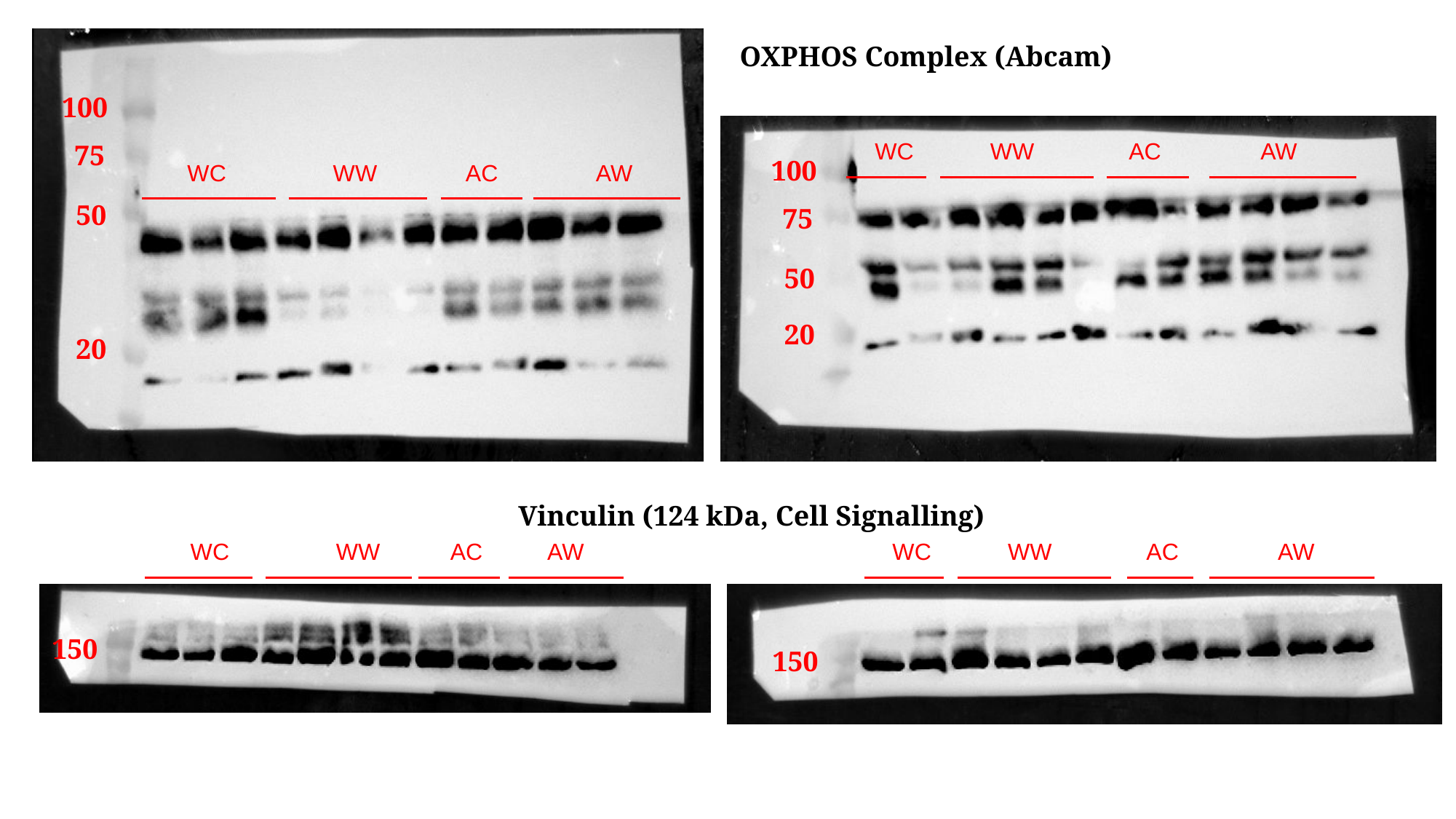

OXPHOS Complex (Abcam)
100
WC
WW
AC
AW
75
100
WC
WW
AC
AW
50
75
50
20
20
Vinculin (124 kDa, Cell Signalling)
WC
WW
AC
AW
WC
WW
AC
AW
150
150
